# Fine-scale evaluation of two standard 16S rRNA gene amplicon primer pairs for analysis of total prokaryotes and archaeal nitrifiers in differently managed soils

**DOI:** 10.3389/fmicb.2023.1140487

**Published:** 2023-02-23

**Authors:** Jun Zhao, Jonathan Rodriguez, Willm Martens-Habbena

**Affiliations:** Fort Lauderdale Research and Education Center, University of Florida, Davie, FL, United States

**Keywords:** 16S rRNA gene amplicon Illumina sequencing, metagenomics, soil, ammonia-oxidizing archaea, primer evaluation, microbial communities, land use change

## Abstract

The advance of high-throughput molecular biology tools allows in-depth profiling of microbial communities in soils, which possess a high diversity of prokaryotic microorganisms. Amplicon-based sequencing of 16S rRNA genes is the most common approach to studying the richness and composition of soil prokaryotes. To reliably detect different taxonomic lineages of microorganisms in a single soil sample, an adequate pipeline including DNA isolation, primer selection, PCR amplification, library preparation, DNA sequencing, and bioinformatic post-processing is required. Besides DNA sequencing quality and depth, the selection of PCR primers and PCR amplification reactions arguably have the largest influence on the results. This study tested the performance and potential bias of two primer pairs, i.e., 515F (Parada)-806R (Apprill) and 515F (Parada)-926R (Quince) in the standard pipelines of 16S rRNA gene Illumina amplicon sequencing protocol developed by the Earth Microbiome Project (EMP), against shotgun metagenome-based 16S rRNA gene reads. The evaluation was conducted using five differently managed soils. We observed a higher richness of soil total prokaryotes by using reverse primer 806R compared to 926R, contradicting to *in silico* evaluation results. Both primer pairs revealed various degrees of taxon-specific bias compared to metagenome-derived 16S rRNA gene reads. Nonetheless, we found consistent patterns of microbial community variation associated with different land uses, irrespective of primers used. Total microbial communities, as well as ammonia oxidizing archaea (AOA), the predominant ammonia oxidizers in these soils, shifted along with increased soil pH due to agricultural management. In the unmanaged low pH plot abundance of AOA was dominated by the acid-tolerant NS-Gamma clade, whereas limed agricultural plots were dominated by neutral-alkaliphilic NS-Delta/NS-Alpha clades. This study stresses how primer selection influences community composition and highlights the importance of primer selection for comparative and integrative studies, and that conclusions must be drawn with caution if data from different sequencing pipelines are to be compared.

## 1. Introduction

Soil harbors high abundance and diversity of prokaryotic microorganisms, with 1 g of soil containing up to 10 billion microbial cells (Torsvik and Øvreås, 2002) and 10^3^–10^6^ phylotypes (Bickel and Or, 2020), including many microorganisms that play critical biogeochemical roles (Crowther et al., 2019). With continuous expansion of prokaryotic diversity and the development of next-generation sequencing tools (Hug et al., 2016), it is important to select a proper pipeline for in-depth analysis of microbial communities. Amplicon-based high throughput sequencing targeting 16S rRNA genes is the most frequently used approach to generating overall profiles of prokaryotic diversity in various environments (Yarza et al., 2014), but the key aspects, e.g., primer design, have been regularly modified and optimized (Fischer et al., 2016; Walters et al., 2016; Fadeev et al., 2021). To facilitate systematic analysis of global microbial taxonomic diversity on the planet and to allow data comparison between different environments and studies, the Earth Microbiome Project (EMP) developed several standard protocols for environmental microbiome analysis, including one for prokaryotic microorganisms using paired-end 16S rRNA gene sequencing on the Illumina platform (Thompson et al., 2017). Currently, two sets of primers for 16S rRNA genes are recommended in the EMP standard pipeline,^1^ i.e., primers 515F–806R for the V4 region (Apprill et al., 2015; Parada et al., 2016), and primers 515F–926R, targeting V4–V5 region (Quince et al., 2011; Parada et al., 2016) of 16S rRNA gene. Both primers have been widely used in previous studies, although *in silico* evaluation of these two primers indicated 515F–926R covers higher prokaryotic diversity than 515F–806R (Parada et al., 2016). However, *in vitro* test using mock community (Fouhy et al., 2016; Parada et al., 2016; Wear et al., 2018) or field samples (Fischer et al., 2016; Thijs et al., 2017; Wear et al., 2018; Fadeev et al., 2021; Bai et al., 2022) is also necessary for comprehensive evaluation of the primer performance. Indeed, it has been demonstrated that *in silico* evaluation of primers does not always reflect *in vitro* performance obtained from experimental tests (Fischer et al., 2016). Especially, evaluating primers using field samples is important in addition to using mock communities, as the complexity is expected to be higher in soils (Rappé and Giovannoni, 2003; Pedrós-Alió and Manrubia, 2016; Lloyd et al., 2018), which could magnify the primer bias toward specific taxa (Eloe-Fadrosh et al., 2016; Parada et al., 2016). The increasing availability of shotgun metagenomes, deemed to be less prone to compositional bias than PCR-based methods, now allows for further ground-truthing of amplicon sequencing approaches (Eloe-Fadrosh et al., 2016).

Cultivation-independent tools have rapidly expanded the understanding of the diversity, evolution and ecology of functional microbial guilds, particularly within the domain of *Archaea*, which also play an important role in sustaining the terrestrial microbial biodiversity and biogeochemistry (Offre et al., 2013; Baker et al., 2020). Importantly, the ammonia oxidizing archaea (AOA) within the archaeal class of *Nitrososphaeria* (previously known as phylum *Thaumarchaeota*) are widespread and often account for the highest proportion of archaea in soil (Bates et al., 2011; Wang et al., 2015; Zhao et al., 2015; Liu et al., 2019), and are critical for terrestrial nitrogen cycling (Gubry-Rangin et al., 2010; Zhang et al., 2010, 2012; Hink et al., 2018; Zhao et al., 2020). Oxygen availability, pH and temperature have been recognized to drive the radiative evolutionary diversification of terrestrial AOA lineages and their differential niche adaptation (Gubry-Rangin et al., 2015; Ren et al., 2019; Yang et al., 2021), leading to a consortium of phylogenetically distinct phylotypes co-existing and competing in the same soil (Gubry-Rangin et al., 2011; Zhao et al., 2015; Huang et al., 2021). Due to the low rate of horizontal gene transfer within *Nitrososphaeria* (Sheridan et al., 2020), the congruence is well supported between genome-resolved phylogeny and some single-copy marker gene phylogenies, including the 16S rRNA gene (Oton et al., 2016; Sheridan et al., 2020; Wang et al., 2021). So far, 12 putative family-level AOA clades can be confidently identified from established 16S rRNA gene database (Wang et al., 2021), and continuous expansion of metagenome assembled genomes (MAGs) of AOA allow characterization of novel lineages using 16S rRNA genes as biomarker. However, there is little information about the potential bias toward specific AOA clade by using different 16S rRNA gene primers.

Environmental disturbances due to anthropogenic activity can drastically alter microbial diversity and ecosystem functions in terrestrial ecosystems (Rillig et al., 2019, 2021). Particularly, the expansion of cropping systems by the conversion of native land will change soil physiochemical properties, leading to shift in microbial diversity and nutrient cycling (Zhou et al., 2020), including nitrogen transformations (Zhao et al., 2020, 2021; Klimasmith and Kent, 2022; Sun et al., 2022). For instance, it is a common practice to modify soil pH to neutral or near-neutral condition for maximum crop productivity, e.g., by liming of acidic forest soil or reclamation of saline-sodic soil (Zhao et al., 2020; Sun et al., 2021). Soil pH is recognized as the main driver for microbial assembly and distribution (Rousk et al., 2010; Tripathi et al., 2018), and the major determinant for niche differentiation of soil AOA (Gubry-Rangin et al., 2011). Other important environmental parameters, such as soil moisture and organic matter, can also be affected by agricultural managements and affect soil microbial distribution and activity (Frindte et al., 2019; Domeignoz-Horta et al., 2021), including ammonia oxidizers (Thion and Prosser, 2014). While correlation analysis is often used to predict the main influencing factors for soil microbial community change after land use conversion, the potential effect of selected primers on identifying these environmental factors remains less clear.

In this study, we examined the performance of the two standard primer pairs targeting the 16S rRNA gene for analyzing total soil microbiome composition and the specific functional guild of nitrifying archaea in soils under different land uses. The 16S rRNA genes retrieved from shotgun metagenome data, which should have no primer-induced bias, were analyzed as a reference point to compare with the amplicon sequencing results.

## 2. Materials and methods

### 2.1. Soil sample collection

A total of 90 soil samples were used for this research. Soils were collected in five different plots under different land uses in the Everglades Agricultural Area (EAA) including an unmanaged native plot (plot 1), a sugarcane-spinach-fallow-sugarcane rotation plot (plot 2), a sugarcane-sweetcorn-rice-sugarcane rotation plot (plot 3), a sugarcane-sweetcorn-fallow-sugarcane rotation plot (plot 4), and a year-round sugar cane plantation plot (plot 5). Plots 2–5 had been historically cultivated with sugar cane for 2 (plot 5) or 3 (plot 2–4) years. Starting from January 2017, plot 2 was planted with spinach till May 2017, followed by a 28-week fallow period before sugar cane plantation in December 2017. Plot 3 was planted with sweet corn from January to May 2017, followed by rice cultivation from May to October 2017 and an 8-week fallow period before sugar cane plantation in December 2017. Plot 4 was planted with sweet corn from January to May 2017, also followed by a 28-week fallow before sugar cane plantation in December 2017. Plot 5 was cultivated with sugar cane for a third consecutive year. Soils were collected during May 2017 to April 2018 at 2–3-month intervals. The area of each plot is ~20,000 m^2^ (~100 × 200 m square). Biological triplicates were sampled in each plot, each containing 9 random cores of 10-cm topsoil within a subplot of ~10 × 10 m square, and the samples were immediately transported to the laboratory on ice. Part of the soil samples were stored in –20°C before DNA extraction.

### 2.2. Soil physiochemistry measurements

Soil pH was measured in slurry with a soil-to-water ratio of 1:2 (10 g of soil in 20 ml deionized water) after shaking for 1 h at room temperature. Soil moisture was determined by calculating weight loss after drying 20 g fresh soil at 105°C for 24 h. Soil ammonium and nitrate were extracted with 2 M KCl by mixing 5 g fresh soil with 45 ml of KCl solution, followed by homogenization on a shaker at 50 rpm for 1 h and centrifuge at 3,000 *g* for 10 min. The supernatant was filtered through a 0.45 μm Nylon filter and the ammonium and nitrate concentrations were then determined colorimetrically following salicylate method (Bower et al., 1980) and VCl_3_-Griess method (García-Robledo et al., 2014), respectively. Total phosphorus (TP) and available phosphorus (AP) concentrations were measured using a continuous segmented flow analyzer (Auto Analyzer 3, Seal Analytical Inc., Mequon, WI, United States) following the EPA method 365.1 (Determination of Phosphorus by Semi-Automated Colorimetry, Revision 2.0). Soil organic matter (OM) content was measured by calculating loss on ignition and dissolved organic carbon (DOC) content was measured using a TOC analyzer (Shimadzu, Kyoto, Japan).

### 2.3. DNA extraction and amplicon sequencing

Soil DNA was extracted using 0.25 g of soil by DNeasy PowerSoil Kit (QIAGEN) following manufacturer’s instruction, except that bead-beating was performed twice using a FastPrep-24 (MP Biomedicals, Santa Ana, CA, United States) at 4.5 m s^−1^ for 15 s. Soil DNA extract was diluted to a concentration of 10 ng μL^−1^ to minimize variability during PCR and stored at-20°C before PCR amplification.

Wet-lab work for amplicon sequencing including PCR amplification, library preparation and DNA sequencing was performed at the Environmental Sample Preparation and Sequencing Facility at Argonne National Laboratory. Amplification of 16S rRNA gene fragments by PCR followed EMP standard pipeline, using either primers 515F–806R (Apprill et al., 2015; Parada et al., 2016) or 515F–926R (Quince et al., 2011; Parada et al., 2016). The forward primer 515F (GTGYCAGCMGCCGCGGTAA) was the same for both primer pairs while the reverse primers were different (806R: GGACTACNVGGGTWTCTAAT and 926R: CCGYCAATTYMTTTRAGTTT), resulting in amplicon size of ~253 and ~374 bp length, respectively. Adapter sequences were fused to the forward primer and reverse primer contained 12 base barcode sequence. PCR reactions consisted of 9.5 μl of DNase-free PCR Water (MoBio, Carlsbad, CA, United States), 12.5 μl of 2x AccuStart II PCR ToughMix (QuantaBio, Beverly, MA, United States), 1 μl of 200 pM forward primer and reverse primer, and 1 μl of template DNA. The PCR thermocycling conditions were as follows: denaturation at 94°C for 3 min, followed by 35 cycles of 94°C for 45 s, 50°C for 60 s, and 72°C for 90 s, and final extension at 72°C for 10 min. PCR products were quantified using PicoGreen (Invitrogen, Carlsbad, CA, United States) in a 96-well microplate reader (Infinite 200 PRO, Tecan, Grödig, Austria). PCR products were pooled in equimolar ratios, purified using AMPure XP Beads (Beckman Coulter, Brea, CA, United States), and quantified using Qubit DNA quantification kit (Invitrogen, Carlsbad, CA, United States). PCR products were diluted to 2 nM for denaturation, and then diluted to 6.75 pM final concentration with 10% PhiX spike. Sequencing was performed on an Illumina MiSeq instrument using 2 × 150 (for 515F-806R PCR products) or 2 × 250 (for 515F-926R PCR products) reagent kit (Illumina, San Diego, CA, United States). The raw 16S rRNA gene amplicon sequencing data were deposited to NCBI under BioProject accession number PRJNA831877.

Paired-end reads were processed by QIIME2 version 2020.11 (Caporaso et al., 2010). The DADA2 plug-in with standard settings was used for quality filtering, denoising, paired-end read merging, chimera removal, dereplication and generation of amplicon sequence variants (ASVs; Callahan et al., 2016). This resulted in 89,103 ± 3,615 and 21,752 ± 529 reads per sample from primers of 515F–806R and 515F–926R, respectively, which were then taxonomically classified against SILVA database release 138.1 (Quast et al., 2013) using the scikit-learn classifier (Pedregosa et al., 2011) embedded in QIIME2. For more detailed taxonomic classification, sequences of ASVs belonging to class *Nitrososphaeria* (excluding non-AOA group 1.1c) were picked out and re-classified into putative family-level clades using BLASTN against a custom AOA database (Wang et al., 2021). A total of 4,302 ± 217 and 1,130 ± 50 reads AOA per sample were obtained from primers of 515F–806R and 515F–926R, respectively.

### 2.4. Metagenome shotgun sequencing

It is expected that the results generated from shotgun metagenome data should not be affected by primer-induced bias and thus can be deemed as proxy of the actual community composition. Therefore, to assess the accuracy of the different primer pairs for analyzing microbial composition in soils, the results of amplicon sequences were compared with 16S rRNA genes obtained from shotgun metagenomes. Shotgun metagenome sequencing was conducted in three of the five soil plots (plot 1, 3 and 5) in July and December of 2017 at the Department of Energy Joint Genome Institute using standard (2 × 151 bp) library preparation and sequencing workflow for Illumina NovaSeq S4 instrument platform and yielded between 80,108,594 ± 4,478,157 per sample high quality metagenome reads (read length > 150 bp, *Q-*score > 30). A total of 12,420 ± 671 16S rRNA gene reads per sample were retrieved from metagenomes by SortMeRNA v4.0.086 (Kopylova et al., 2012). These sequences were then dereplicated using “qiime vsearch dereplicate-sequences” plug-in in QIIME2 and the representative sequences were taxonomically classified against SILVA database using the scikit-learn classifier as described above. AOA sequences were extracted and reclassified by BLASTN against the same custom AOA database as used for analyzing amplicon sequencing data (see above). Metagenomic shotgun sequences are deposited at NCBI under SRA study IDs SRP258623, SRP258632, SRP258633, SRP258644, SRP258647, SRP258661, SRP258662, SRP258690, SRP258692, SRP258713, SRP258714, SRP258717, SRP258719, SRP258720, SRP258807, SRP258809, SRP258810, SRP270154.

### 2.5. Statistical analyses

Alpha diversity (number of ASVs and Pielou’s evenness) was calculated after normalization of the sequences to the depth of 12,000 reads per sample. Principal coordinates analysis (PCoA) of weighted UniFrac distance matrices was performed using the vegan package v2.5–7 (Dixon, 2003) in R version 4.1.1, and permutational multivariate analysis of variance (PERMANOVA) was used to assess the compositional difference between different land uses and primer pairs. To characterize the correlation of environmental variables and microbial community compositions, the ‘envfit’ function of the vegan package was used to test the significance of eight soil physiochemical properties (soil pH, OM, moisture, DOC, TP, AP, nitrate and ammonium concentrations) for the PCoA ordinations. Differences at *p* < 0.05 were considered statistically significant.

The proportion of each taxon in a soil was calculated. We selected the major phyla (with average proportion > 0.5% in all soils by amplicon sequencing) to evaluate the performance of the primers. For each major phylum identified by both primer pairs, the proportions were compared against each other using a paired sample *t*-test across all samples from each soil plot, as similarly used previously (Toole et al., 2021). We further calculated the proportion of each AOA clade relative to total AOA sequences, and performed paired sample *t*-test to compare the proportions by using different primer pairs. Differences at *p* < 0.05 were considered statistically significant.

To evaluate the accuracy of amplicon sequencing for estimating the proportion of a specific taxon in a plot, we calculated log_2_-transformed ratios by dividing the proportion of the taxon from amplicon sequencing data (from 515F-806R or 515F-926R) by the proportion within each sample’s respective metagenomome-16S rRNA, as similarly used previously (Wear et al., 2018). The absolute values of log_2_ ratios between 515F-806R and 515F-926R were compared with each other using paired sample *t*-test across all samples in each soil plot. Differences at *p* < 0.05 were considered statistically significant and a significantly smaller absolute value indicated a more accurate estimation of proportion compared to metagenome result.

The *R* package “ggplot2” (Wickham, 2016) was used to plot the results of diversity indices and relative abundances of different taxa. The log_2_ ratio results were plotted using OriginPro 2016.^2^ And Adobe Illustrator 2019 was used to merge different figure plots.

### 2.6. *In silico* evaluation of the primer coverage

The theoretical coverage of the two primer pairs for analyzing total prokaryotes was tested *in silico* by using the TestProbe function of the SILVA database 138.1 server.^3^ Additionally, the primers were also evaluated for the coverage of specific AOA clades by Probe Match function of ARB (Ludwig et al., 2004) against the AOA 16S rRNA gene database after removing reads that do not contain the primer locus region (Wang et al., 2021).

## 3. Results

### 3.1. *In silico* evaluation of the two primer pairs

The *in silico* evaluation was in agreement with previous results (Parada et al., 2016). Primers 515F-926R resulted in higher coverage of the total database sequences, as well as higher coverage of the sequences from all domains and the common phyla (Table 1). Only *Crenarchaeota* and *Firmicutes* were consistently less represented by 515F-926R compared to 515F-806R. Primers 515F-926R also resulted in less coverage of *Entotheonellaeota* than using 515F-806R when perfect match was required, but this trend was reversed when one mismatch was allowed. We also tested the primer coverage to a well classified AOA 16S rRNA database (Wang et al., 2021) and found both primer pairs matched perfectly to all sequences (102 sequence) in the database. The degeneracy of the primers did not affect the 515F or 806R matching, as a single primer variant was responsible for the perfect match in both primer binding sites. However, 926R had 101 perfect matches with one specific primer sequence and one perfect match with a different primer sequence, showing that the degeneracy was necessary.

**Table 1:** *In silico* assessment of the coverage (percentage of matched sequences) against SILVA SSU 138.1 database of the two different primer pairs 515F-806R or 515F-926R, allowing zero or one mismatch, respectively.

	0 mismatch		1 mismatch	
	515F-806R	515F-926R	515F-806R	515F-926R
Total taxa in SILVA database	75.6%	85.1%	83.2%	93.6%
Domain-level taxa				
*Archaea*	83.5%	81.0%	94.6%	90.8%
*Bacteria*	83.6%	84.5%	92.8%	93.4%
*Eukaryota*	0.1%	80.8%	2.0%	91.4%
Phylum-level taxa				
*Proteobacteria*	87.9%	89.4%	93.5%	94.3%
*Acidobacteriota*	90.7%	92.7%	95.5%	97.1%
*Actinobacteriota*	78.6%	82.1%	91.8%	92.8%
*Chloroflexi*	56.6%	79.4%	87.7%	94.5%
*Planctomycetota*	81.9%	85.0%	94.1%	95.7%
*Bacteroidota*	86.9%	88.3%	94.7%	94.8%
*Crenarchaeota*	85.4%	83.4%	95.8%	90.3%
*Myxococcota*	90.6%	91.9%	96.0%	97.5%
*Verrucomicrobiota*	79.2%	82.4%	94.4%	95.0%
*Gemmatimonadota*	88.2%	92.3%	93.5%	96.5%
*Methylomirabilota*	88.9%	93.0%	93.8%	98.4%
*Firmicutes*	84.4%	83.6%	92.0%	91.9%
*Nitrospirota*	89.6%	91.5%	95.3%	96.5%
*Entotheonellaeota*	88.2%	75.7%	95.4%	99.3%
*Desulfobacterota*	87.4%	89.8%	94.9%	95.5%
*Cyanobacteria*	75.7%	85.2%	89.0%	92.0%
*Latescibacterota*	89.7%	90.1%	95.4%	95.8%

The assessment was conducted within three domains and 17 major phyla characterized in the soils tested in this study.

### 3.2. Comparison of total prokaryotic communities from 16S rRNA amplicon sequences using different primer pairs

**Figure 1:**
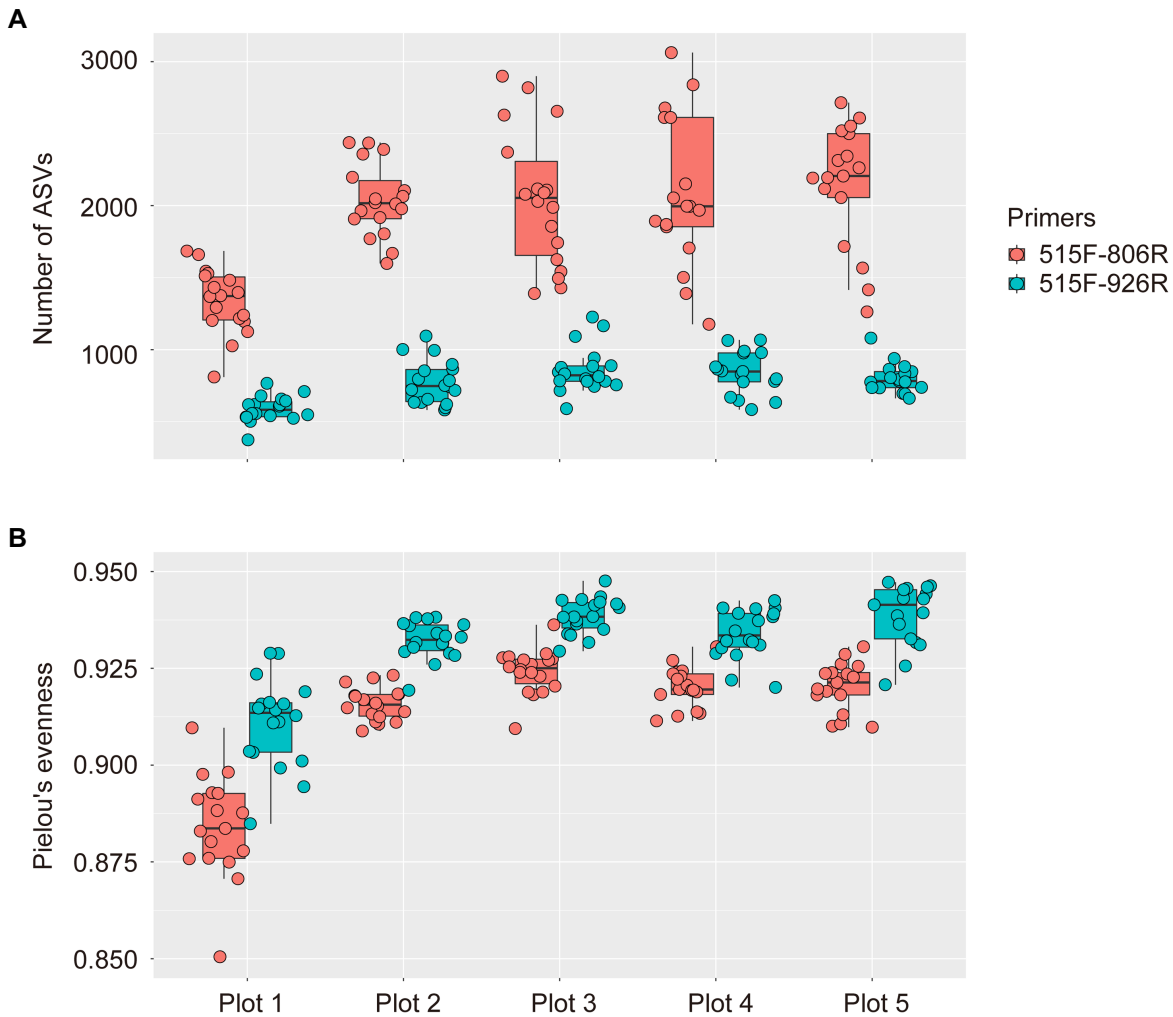
Box plots showing the alpha diversity of soil prokaryotes in five differently managed plots from the Everglade Agricultural Area based on high-throughput sequencing of 16S rRNA genes amplified with primer pairs 515F-806R or 515F-926R. **(A)** The number of ASVs reflecting richness of species. **(B)** Pielou’s evenness index of prokaryotes. The upper and lower bounds of boxes correspond to the 25th and 75th percentiles, with a median line shown. Whiskers denote the 1.5 IQR (interquartile range) and dots represent individual samples.

To our surprise, much higher microbial richness (indicated by number of ASVs) was observed in all soil plots by using 515F-806R (1,926 ± 52 ASVs, MEAN ± SE) compared to that from 515F-926R (772 ± 17; Figure 1), after sequencing 90 soil samples from the five differently managed plots in EAA. In contrast, higher evenness of microbial communities (indicated by Pielou’s evenness index) was detected by 515F-926R (0.931 ± 0.001) than using 515F-806R (0.912 ± 0.002). Despite the difference in alpha diversity of microbial communities detected by these two primer pairs, both captured significantly lower richness of microbes in the unmanaged plot (plot 1) compared to those in the agriculturally managed plots (plot 2–5) (*p* < 0.001; Figure 1).

**Figure 2:**
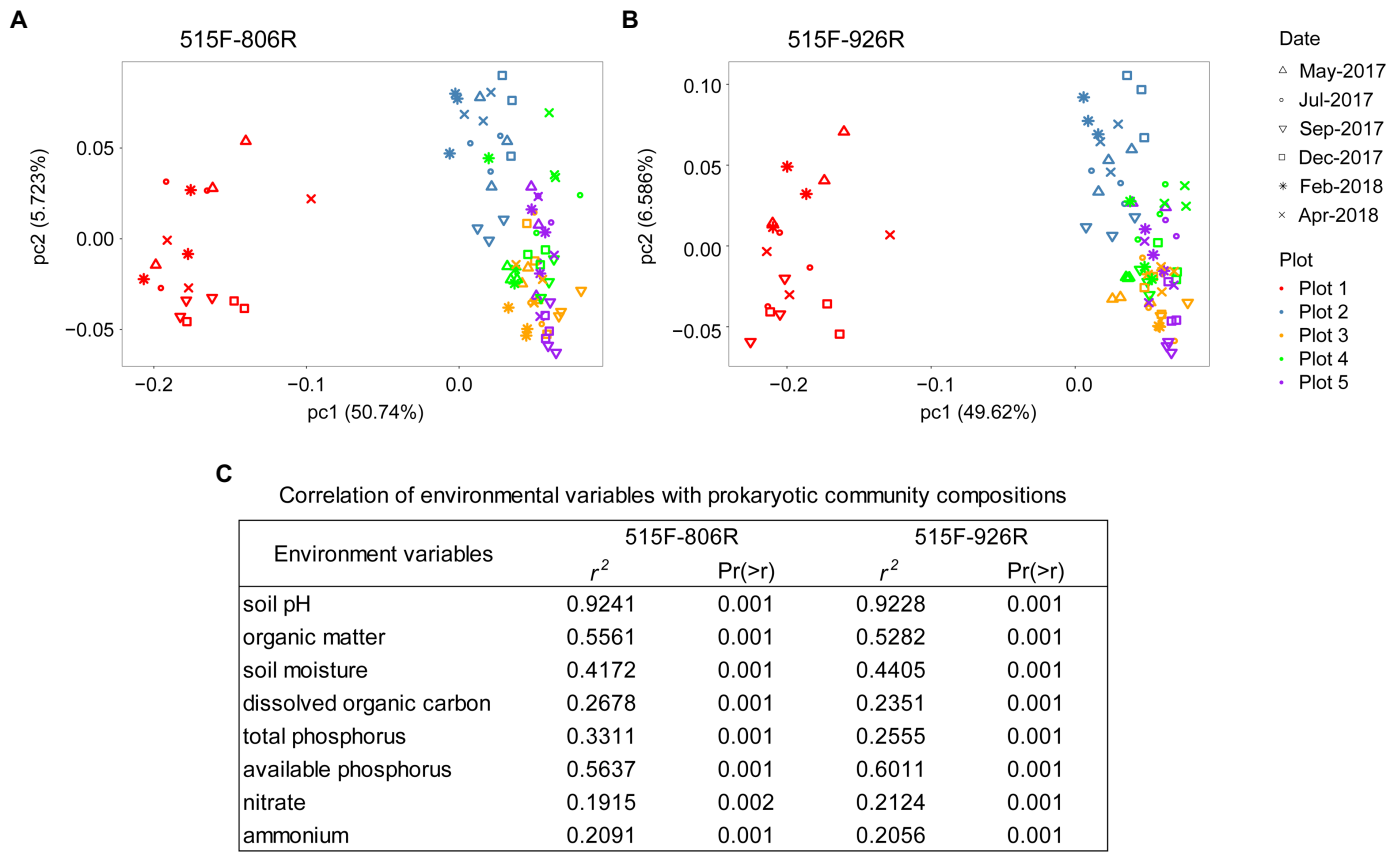
PCoA plot of prokaryotic composition based on the ASVs generated from 16S rRNA genes based on primer sets 515F-806R **(A)** or 515F-926R **(B)**, and correlation between microbial community variation and environmental variables **(C)**.

Different primer pairs can affect the characterization of microbial composition in the soils (Supplementary Figure S1). However, PCoA on the unweighted UniFrac distance matrix revealed the same pattern of variation in microbial composition between different plots (Pairwise PERMANOVA, *p* < 0.001 between any two plots), irrespective of primer pair used (Figure 2). All eight environmental variables tested in this study (data in Supplementary Table S1) showed significant correlation with the variation in microbial composition among different soils (*p* < 0.002; Figure 2). The results from both primer pairs indicated that soil pH had the strongest correlation (*r*^2^ > 0.92) with the microbial composition variation, followed by available phosphorus and organic matter content. RDA on species distribution consistently characterized the pH as the dominant environmental variables explaining majority of the microbial variations between different plots (806R: 63.7%, pseudo-*F* = 124, *p* = 0.002; 926R: 55.9%, pseudo-*F* = 90.2, *p* = 0.002), while other environmental variables explained much less (all ≤3.7%, see Supplementary Table S2).

**Figure 3:**
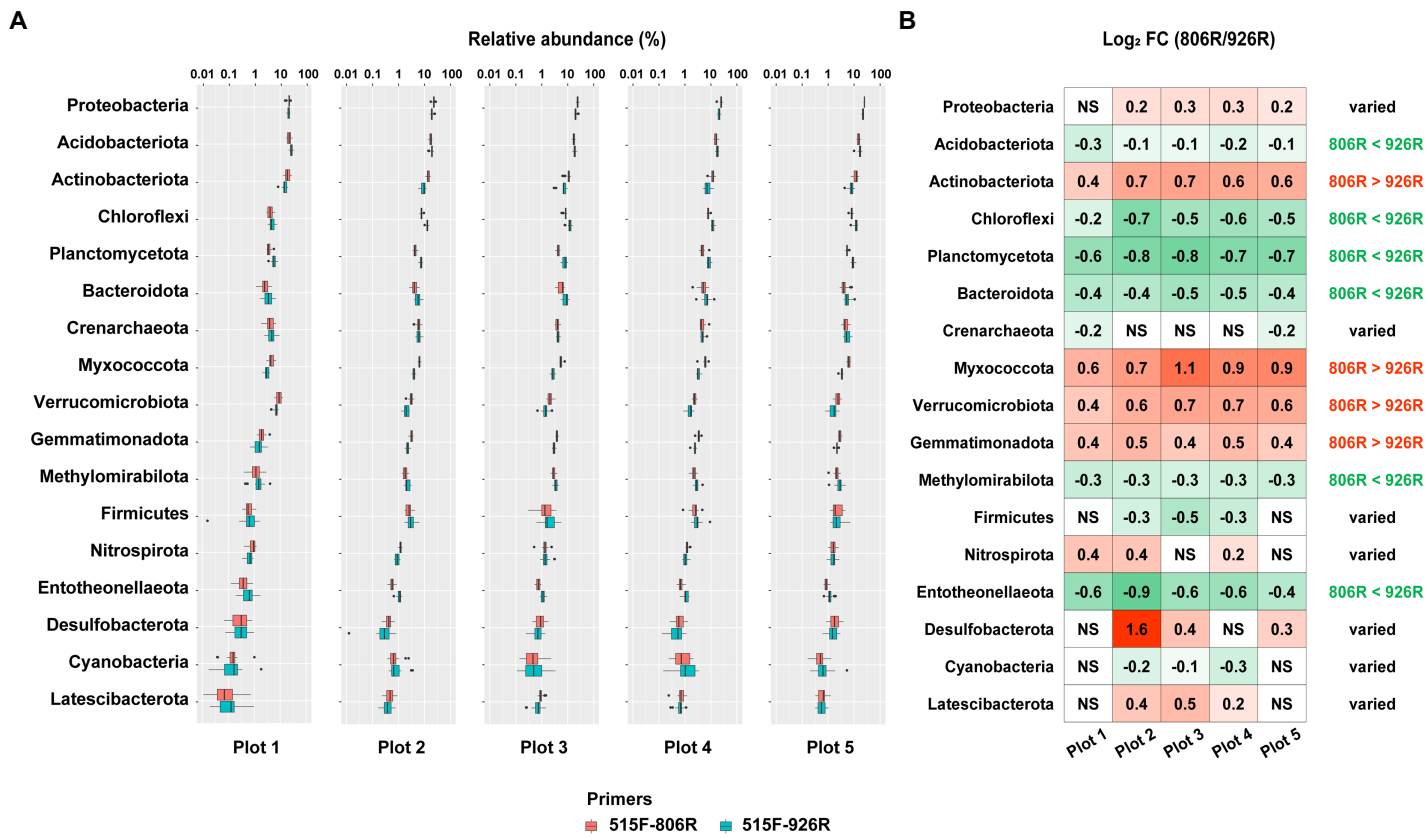
The comparison of estimated relative abundances of major phyla in the soils using different primers. **(A)** The relative abundance of top 17 most abundant phyla (>0.5% on average) in five differently managed plots from the Everglade Agricultural Area based on high-throughput sequencing of 16S rRNA genes amplified using primer pairs 515F-806R or 515F-926R. **(B)** The log_2_-transformed fold change in the relative abundances by using 515F-806R compared to using 515F-926R. A positive value indicates significantly higher relative abundance by using 515F-806R than 515F-926R; a negative value indicates significantly higher relative abundance by using 515F-926R than by 515F-806R; and “NS” indicates the same relative abundances by using these two primer pairs. Generalization of the patterns from the five plots is shown in the rightmost column, with “806R > 926R” indicating higher relative abundance by using 515F-806R in all plots, “806R < 926R” indicating higher relative abundance by using 515F-926R in all plots. “Varied” refers to no consistent bias observed in different plots.

To provide detailed information of the effect of different primer pairs on assessing the relative abundance of the major taxa, we selected the commonly detected phyla in soils (with average relative abundance >0.5% in amplicon sequencing data) and compared their proportions by using different primer pairs. A total of 17 phyla were selected (Figure 3A; Supplementary Figure S2), among which four (*Actinobacteriota*, *Myxococcota*, *Verrucomicrobiota*, *Gemmatimonadota*) showed consistently higher proportions in the 515F-806R data, than in the 515F-926R data, and six phyla (*Acidobacteriota*, *Chloroflexi*, *Planctomycetota*, *Bacteroidota*, *Methylomirabilota*, *Entotheonellaeota*) showed the opposite trend (Figure 3B). The other seven phyla (*Proteobacteria*, *Crenarchaeota*, *Firmicutes*, *Nitrospirota*, *Desulfobacterota*, *Cyanobacteria*, *Latescibacterota*) showed inconsistent patterns of proportions using 515F-806R versus 515F-926R primer pairs, depending on soil management. For instance, 515F-806R resulted in significantly higher proportion of *Proteobacteria* in the agricultural soil plots 2–5, but the proportion of *Proteobacteria* did not change in the unmanaged plot 1, compared to the results obtained from 515F-926R.

### 3.3. Comparison of AOA communities from 16S rRNA gene amplicon sequences using different primer pairs

Ammonia oxidizing archaea represented the dominant archaea in all our soils, irrespective of primers used. The proportion of AOA relative to total archaeal reads ranged between 92.3% ± 1.3 to 99.2% ± 0.2% and between 90.4% ± 1.4 to 99.7% ± 0.2% in different plots by using primer pairs of 515F-806R and 515F-926R, respectively. Both primer pairs also resulted in similar proportions of AOA relative to total prokaryotes, ranging between 4.0% ± 0.1 to 6.0% ± 0.3% and between 4.1% ± 0.1 to 6.2% ± 0.3% in different plots by using 515F-806R and 515F-926R, respectively. As expected, ammonia oxidizing bacteria (AOB) were rarely detected in these soils (proportion ≤ 0.08% and ≤ 0.05% relative to total prokaryotes using 515F-806R and 515F-926R, respectively), since no chemical nitrogen fertilizer was applied to stimulate AOB growth and activity (Verhamme et al., 2011; Tao et al., 2021b,c).

**Figure 4:**
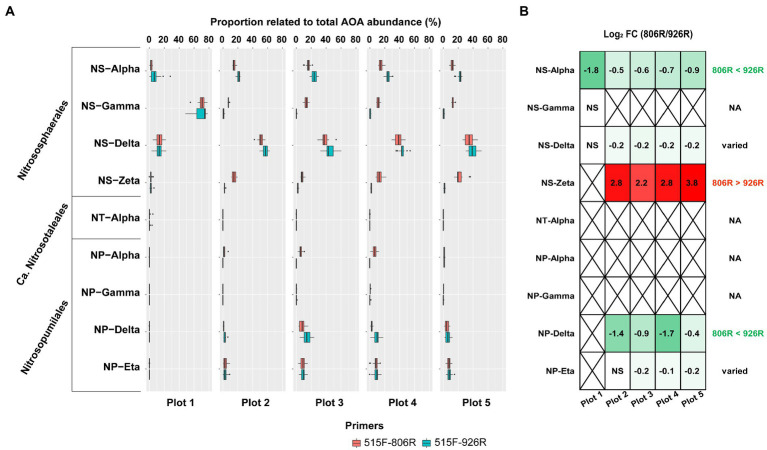
The comparison of estimated proportion of AOA clades in the soils using different primers. **(A)** The proportion of the nine AOA clades relative to all AOA reads in five differently managed plots from Everglade Agricultural Area based on high-throughput sequencing of 16S rRNA genes amplified with primer pairs 515F-806R or 515F-926R. **(B)** The log_2_-transformed fold change of relative abundances using 515F-806R compared to using 515F-926R. A positive value indicates significantly higher relative abundance characterized by using 515F-806R than 515F-926R; a negative value indicates significantly higher relative abundance using 515F-926R comparted to using 515F-806R; “NS” indicates the same relative abundances by using these two primer pairs; and a cross symbol means statistics cannot be performed due to absence of related reads in most of the samples. Generalization of the patterns from the five plots is shown in the rightmost column, with “806R > 926R” indicating higher relative abundance by using 515F-806R in all plots, “806R < 926R” indicating higher relative abundance by using 515F-926R in all plots, and “varied” meaning no consistent bias was observed in different plots.

All AOA sequences were classified into putative family-level clades based on a highly resolved phylogeny (Alves et al., 2018; Wang et al., 2021). A total of nine AOA clades were identified in our soils by 16S rRNA gene amplicon sequences (Figure 4A). Four of the AOA clades (NS-Alpha, NS-Delta, NS-Gamma and NS-Zeta) belonging to the order *Nitrososphaerales* constituted the majority of AOA in the tested soils. We also observed four *Nitrosopumilales*-related AOA clades accounting for smaller portions, and very rarely a *Ca.* Nitrosotaleales clade (NT-alpha).

Different primer pairs led to significant change in relative proportion of some major AOA clades relative to total AOA abundance (Figure 4B). For example, primers 515F-926R resulted in higher proportions of NS-Alpha and NP-Delta clades, compared to results from 515F-806R. In contrast, 515F-806R generated consistently higher proportion of NS-Zeta. For characterizing two of the clades, i.e., NS-Delta and NP-Eta, the effect of the different primer pairs was not consistent when used in different soils. The *Ca.* Nitrosotaleales clade (NT-Alpha) was rarely detected by both primer sets.

### 3.4. Comparison of microbial communities from 16S rRNA gene amplicon sequences and shotgun metagenomes

**Figure 5:**
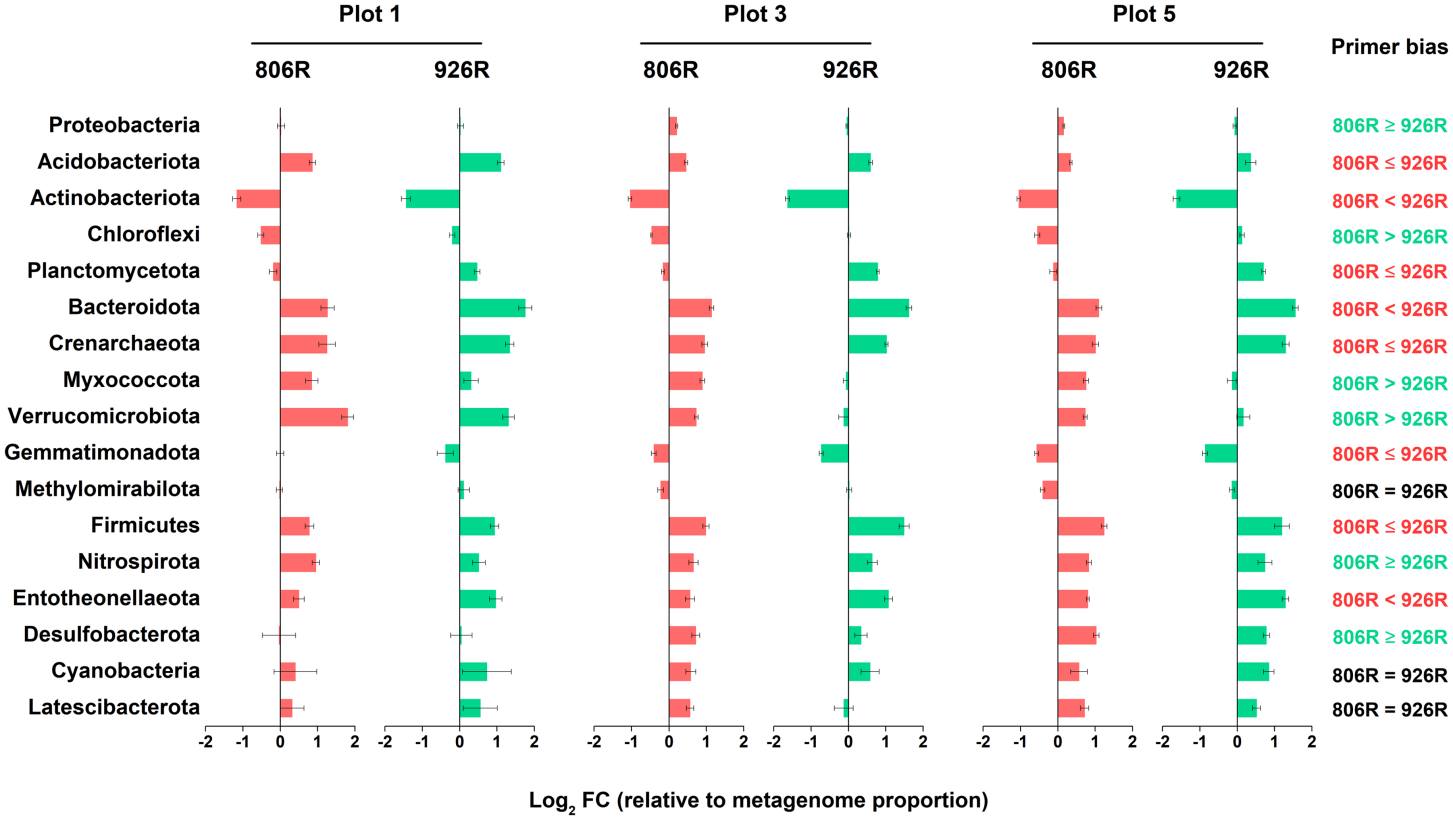
The log_2_-transformed fold change in amplicon-based sequencing results (using 515F-806R or 515F-926R) relative to metagenome-derived 16S rRNA genes. Labels on the right column show generalization of primer bias as compared to metagenome-derived 16S rRNA genes. “806R < 926R” and “806R ≤ 926R” indicate 515F-806R results have less bias and more accurately estimate the proportion of the phyla; “806R > 926R” or “806R ≥ 926R” indicate 515F-926R results have less bias and more accurately estimate the proportion of the phyla, and “806R = 926R” means both primer pairs have same degree of bias toward the phyla.

Shotgun metagenomes (total 18 metagenomic datasets, including replicates) were performed in 3 of the plots (plot 1, 3, and 5) in two time points (July and December of 2017). Metagenomic results showed that some taxonomic patterns were comparable to the amplicon-based sequencing results (Figure 5). However, there was variation in relative abundance by amplicon-based sequencing compared to that from metagenome data. Among all 17 major phyla in the soils, the relative abundances of the metagenome-16S rRNA gene results of eight phyla, i.e., *Acidobacteriota*, *Actinobacteriota*, *Planctomycetota*, *Bacteroidota*, *Crenarchaeota*, *Gemmatimonadota*, *Firmicutes*, and *Entotheonellaeota*, were better approximated by 515F-806R, than by 515F-926R primers. In contrast, the results from 515F-926R primers were more similar to metagenomic results for *Proteobacteria*, *Chloroflexi*, *Myxococcota*, *Verrucomicrobiota*, *Nitrospirota*, and *Desulfobacterota*. No significant differences were observed in the relative abundances of *Methylomirabilota*, *Cyanobacteria*, and *Latescibacterota* by using different primer pairs and shotgun sequencing approaches. We further evaluated the accuracy of different primers for characterizing the major genera (average proportion > 0.5%). Most of the phylum-level patterns also applied to the affiliated genera (Supplementary Figure S3). However, genera from the phyla *Actinobacteriota* and *Chloroflexi*, as well as some genera from *Proteobacteria* and *Acidobacteriota* showed opposite patterns compared to the respective phylum-level results. For instance, the proportions of the four major genera from *Chloroflexi* by 515F-806R were more similar to metagenomic results.

**Figure 6:**
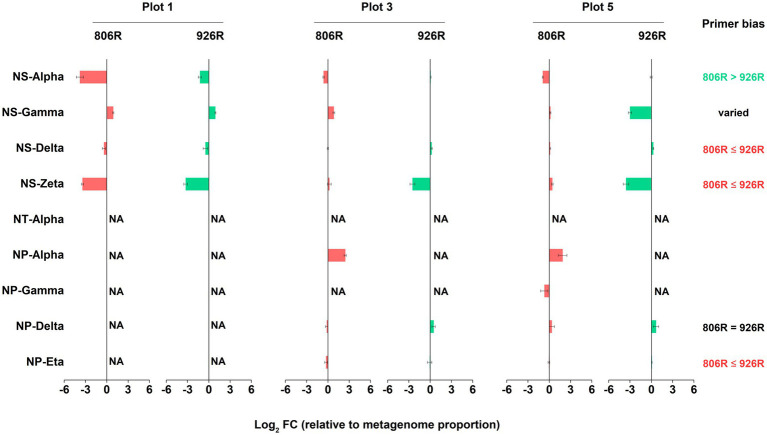
The log_2_-transformed fold change in amplicon-based sequencing results of AOA (using 515F-806R or 515F-926R) relative to metagenome-derived 16S rRNA genes. “NA” means data not available due to lack of sequences in metagenome-derived 16S rRNA gene reads. Labels on the right column show generalization of primer bias as compared to metagenome-16S rRNA results. “806R ≤ 926R” indicates 515F-806R results have less bias and more accurately estimate the proportion of the AOA clades, “806R > 926R” indicates 515F-926R results have less bias and more accurately estimate the proportion of the AOA clade, and “806R = 926R” means both primer pairs have same degree of bias toward the clade.

Similarly, we assessed the accuracy of amplicon-based sequencing results of AOA, in comparison to metagenome-16S rRNA gene results. Due to lack of reads of some AOA clades in the metagenome-16S rRNA gene data, we can only evaluate the results of six AOA clades (Figure 6). We found 515F-806R results of NS-Delta, NS-Zata and NP-Eta had higher similarity to metagenome results, while 515F-926R showed more accurate results of NS-Alpha. The effect of these two primer pairs on estimation of NP-Delta proportion was the same, but the effect on NS-Gamma proportion was not consistent in different soils.

## 4. Discussion

One of the major criteria for selecting primers is their coverage of target microbes. In this study, the two primer pairs yielded significantly different alpha diversity. Particularly, 515F-806R resulted in higher richness of microorganisms compared to 515F-926R, as reflected by higher numbers of ASVs (Figure 1A). This result contradicted the *in silico* assessment using the most updated SILVA database, in which 515F-926R appeared to cover higher diversity of total prokaryotes. The incongruence between *in silico* and *in vitro* results has been observed previously (Fischer et al., 2016; Pausan et al., 2019). As the primers were used to generate profiles of highly complex microbial communities in soils, such inconsistence is most likely due to the various degrees of primer-template mismatches. For instance, the binding regions of 16S rRNA genes of some major prokaryotic taxa might have less mismatches to 926R than to 806R, resulting in over-representation of certain taxa during PCR amplification and consequently an overall decline in richness. Indeed, the presence of a single primer-template mismatch can affect the efficiency of PCR amplification and lead to underestimation of gene numbers by more than 1,000 times, depending on the primer and position of the mismatch (Bru et al., 2008). This will particularly affect the characterization of rare taxa in soils, if they have a high number of mismatches, or the mismatches are particularly enriched near the primer extension sequence (Bru et al., 2008). Rare taxa are pervasive in microbial biosphere and often consist of a large portion of microbial diversity in various environments (Curtis et al., 2002; Lynch and Neufeld, 2015), such as soils used in this study. We cannot rule out the possibility that the differences in the hypervariability of V4 and V4-V5 regions covered by these two primers might resulted in different taxonomic resolutions and thus affected the classification accuracy (Guo et al., 2013; Yang et al., 2016; Kerrigan et al., 2019).

Compared to using mock community, the biggest challenge for primer evaluation using environmental sample is the approach to producing reference results. Methods such as fluorescence *in situ* hybridization (Fadeev et al., 2021) and shotgun metagenome sequencing (Tremblay et al., 2015; Eloe-Fadrosh et al., 2016; Fischer et al., 2016) have been used previously to establish reference datasets. This study used shotgun metagenome-derived 16S rRNA gene data as reference which should generate a more accurate microbial profile than amplicon-based sequencing approaches (Brooks et al., 2015). Through the comparison between amplicon and metagenome results in five differently managed plots (Figure 5), we generalized that: (i) For most major phyla the bias was more pronounced by either 515F-806R or 515F-926R; only for *Methylomirabilota*, *Cyanobacteria*, and *Latescibacterota* the bias was similar between these two primer pairs; (ii) Both primer pairs consistently overestimated relative abundances of phyla *Acidobacteriota*, *Bacteroidota*, *Crenarchaeota*, *Firmicutes*, *Nitrospirota*, and *Entotheonellaeota*, and underestimated *Actinobacteriota*; (iii) The phylum-level evaluation did not always apply to the affiliated genera. The discrepancy between phylum and genus-level results thus emphasizes the necessity of preliminary test of newly acquired environmental samples for accurate quantification of the proportion of specific taxa.

Although the amplicon sequencing of field samples generated inconsistent results between *in silico* evaluation and metagenome-based data for some specific taxa, the use of different primer pairs did not affect the generalization of overall trends of microbial community variations associated with different land uses. For instance, both primer pairs revealed significantly higher richness of soil prokaryotes in agriculturally managed soils (plot 2–5) than in the unmanaged native soil (plot 1, Figure 1A). We also observed significant distinction in microbial composition between different plots irrespective of primer used, especially between unmanaged soil and agricultural soils (Figure 2). Despite primer-induced variation in relative abundance of many taxa, the general patterns of the major phyla abundance can still be well reflected. For instance, both primer pairs showed that the 17 major phyla characterized in our soils collectively predominated the microbial community abundances, accounted for 93.0–99.3% and 94.9–99.5% of the total prokaryotic 16S rRNA reads, respectively (Figure 3). For AOA analysis, both primer pairs showed the highest abundance of NS-Gamma in unmanaged soil, while NS-Delta, NS-Alpha, NP-Delta and NP-Eta co-dominated AOA abundance in agricultural soil (Figure 4).

The conversion of native high-organic matter Everglades soil for agricultural use led to changes in soil physiochemistry and microbiology, and the choice of primers did not affect the revelation of the major environmental factors associated with the microbial community changes. Soil pH is characterized as the primary factor influencing the whole community composition (Figure 2). The unmanaged soil had a pH value between 5.0–5.8 all year round, while the agricultural soils all showed more neutral pH of 6.5–7.8 due to previous liming, leading to drastic difference in prokaryotic community composition between these two types of land uses (Figure 2). This was consistent with previous meta-analysis showing soil pH as the major determinant for microbial assembly process (Tripathi et al., 2018). Soil pH was also recognized as the main driver determining the niche specialization of terrestrial AOA at global scale (Gubry-Rangin et al., 2011) in addition to bacterial nitrifiers (Tao et al., 2021a), and might similarly affect the AOA composition in our soils. Indeed, we found that the unmanaged soil with low pH contained much higher proportion of NS-Gamma AOA clade (Figure 4), which has been classified as acido-neutral clade and can well adapt to acidic soil (Wang et al., 2019; Zhao et al., 2021). As expected, we observed increased proportions of NS-Delta and NS-Alpha in agricultural soils with higher pH, which represent the major AOA clades in soils globally and are adapted to neutral-alkaline pH soil (Gubry-Rangin et al., 2011; Alves et al., 2018; Zhao et al., 2021). Surprisingly, a fair proportion of *Nitrosopumilales*-related AOA clades were also detected in agricultural soils, mostly from clades of NP-Delta and NP-Eta. These two AOA clades are represented by *Nitrosopumilus-*, *Ca*. Nitrosoarchaeum-, and *Ca*. Nitrosotenuis-affiliated strains and were more frequently detected in oligotrophic environments such as marine and deep oligotrophic soil (Mosier et al., 2012; Jung et al., 2014; Tolar et al., 2020). Their presence in the fertile agricultural soils suggested that some of the phylotypes from these clades can adapt well to environments with high nutrient availability. However, the absence of *Nitrosopumilales* AOA in the unmanaged soil implied high sensitivity of related clades to low soil pH.

## 5. Conclusion

This study comprehensively evaluated two commonly used primer pairs for characterization of complex microbial communities in soils, in comparison to *in silico* evaluation and shotgun metagenome-based results. As previously observed, the *in silico* evaluation cannot be confidently used to predict the *in vitro* results of PCR amplification, and we observed lower richness of prokaryotic microorganisms from 515F-926R which theoretically covers higher prokaryotic diversity. It is thus our recommendation that 515F-806R is better for characterization of microbial diversity in organic-rich agricultural soils similar to those used in this study but a preliminary test should be conducted for analyzing microbes in other types of soils (e.g., forest, sediments). Despite dissimilar results by using different primer pairs, the main patterns of microbial community structure (diversity and association to environmental factors) between different soils can still be consistently characterized regardless of primer used. Therefore, it is methodologically rational to integrate amplicon sequencing data from different studies for comparative studies or meta-analyses if the primers were identical, and results should be interpreted with caution if different primer pairs or sequencing pipelines were applied.

## Data availability statement

The datasets presented in this study can be found in online at: https://www.ncbi.nlm.nih.gov/genbank/ under Bioproject PRJNA831877, and https://www.ncbi.nlm.nih.gov/sra under accessions SRP258623, SRP258632, SRP258633, SRP258644, SRP258647, SRP258661, SRP258662, SRP258690, SRP258692, SRP258713, SRP258714, SRP258717, SRP258719, SRP258720, SRP258807, SRP258809, SRP258810, SRP270154.

## Author contributions

JZ and WM-H designed the study. JZ and JR conducted the experiments. JZ drafted the manuscript with input from JR and WM-H. All authors contributed to the article and approved the submitted version.

## Funding

Funding for this project was provided by the Florida Agricultural Experiment Station Hatch project FLA-FTL-005680, UF IFAS Early Career Award, and USDA NIFA award #2022-67019-36501, and Department of Energy Joint Genome Institute Community Sequencing Program Project #503337 to WM-H. The work conducted by the U.S. Department of Energy Joint Genome Institute, a DOE Office of Science User Facility, is supported by the Office of Science of the U.S. Department of Energy under Contract No. DE-AC02-05CH11231.

## Conflict of interest

The authors declare that the research was conducted in the absence of any commercial or financial relationships that could be construed as a potential conflict of interest.

## Publisher’s note

All claims expressed in this article are solely those of the authors and do not necessarily represent those of their affiliated organizations, or those of the publisher, the editors and the reviewers. Any product that may be evaluated in this article, or claim that may be made by its manufacturer, is not guaranteed or endorsed by the publisher.

## Footnotes

^1^ https://earthmicrobiome.org/protocols-and-standards/16s/

^2^ https://www.originlab.com/origin

^3^ https://www.arb-silva.de/search/testprobe/
