# Supplementary material for: Fine-scale evaluation of two standard 16S rRNA gene amplicon primer pairs for analysis of total prokaryotes and archaeal nitrifiers in differently managed soils
Source: Front Microbiol. 2023 Feb 23;14:1140487. doi: 10.3389/fmicb.2023.1140487 (PMC9995467; doi:10.3389/fmicb.2023.1140487)
Supplement: Supplementary file 1 [file Data_Sheet_1.PDF]

Supplementary Material

Fine-scale evaluation of two standard 16S rRNA gene amplicon primer pairs for analysis of total prokaryotes and archaeal nitrifiers in differently managed soils

Jun Zhao[1], Jonathan Rodriguez[1], Willm Martens-Habbena[1*]

[1] Fort Lauderdale Research and Education Center, Department of Microbiology and Cell Science, University of Florida, Davie, FL, 33314, USA

*** Correspondence:**
Willm Martens-Habbena
w.martenshabbena@ufl.edu

Supplementary Table 1. The physiochemical properties of soil from the five differently managed plots.

Plot	pH	Moisture (%)	Organic matter (g kg^{-1} dry soil)	Dissolved organic C (mg kg^{-1} dry soil)	Total P (mg kg^{-1} dry soil)	Available P (mg kg^{-1} dry soil)	Nitrate-N (mg kg^{-1} dry soil)	Ammonium -N (mg kg^{-1} dry soil)
May-17								
1	5.2 ± 0.1	36.9 ± 0.3	822 ± 10	6963 ± 411	1307 ± 88	27.7 ± 3.5	233.6 ± 20.5	21.4 ± 5.0
2	6.7 ± 0.0	49.0 ± 0.3	612 ± 79	3084 ± 86	2279 ± 379	108.0 ± 6.8	83.4 ± 12.9	3.0 ± 0.4
3	7.0 ± 0.1	58.7 ± 0.9	776 ± 13	3579 ± 262	1337 ± 77	54.7 ± 10.1	94.1 ± 12.5	5.4 ± 0.9
4	7.3 ± 0.1	54.7 ± 0.8	728 ± 4	4459 ± 120	1670 ± 101	98.7 ± 9.1	89.3 ± 4.2	3.2 ± 0.2
5	7.5 ± 0.1	58.7 ± 0.8	707 ± 11	4551 ± 296	1656 ± 75	84.0 ± 8.4	16.8 ± 8.1	4.0 ± 0.6
July-17								
1	5.5 ± 0.0	42.8 ± 1.7	827 ± 10	7787 ± 376	1270 ± 47	25.3 ± 0.9	41.5 ± 10.9	5.5 ± 0.4
2	7.2 ± 0.0	51.0 ± 0.3	700 ± 10	3505 ± 194	2275 ± 68	101.0 ± 6.7	16.5 ± 1.6	1.9 ± 0.2
3	7.3 ± 0.0	66.4 ± 1.3	783 ± 4	3464 ± 322	1362 ± 48	60.0 ± 3.1	0.4 ± 0.2	2.1 ± 0.2
4	7.5 ± 0.0	55.3 ± 0.4	737 ± 2	4991 ± 316	1649 ± 89	98.7 ± 5.9	5.8 ± 2.6	2.1 ± 0.0
5	7.7 ± 0.0	55.8 ± 0.4	677 ± 4	5373 ± 311	2236 ± 251	103 ± 11.9	1.0 ± 0.1	2.9 ± 0.1
Sep-17								
1	5.5 ± 0.1	51.6 ± 0.3	830 ± 4	8522 ± 154	1270 ± 70	24.7 ± 2.6	30.6 ± 2.8	4.0 ± 0.3
2	7.2 ± 0.0	55.0 ± 1.5	735 ± 42	4681 ± 57	1769 ± 340	122.3 ± 0.9	7.8 ± 0.5	1.9 ± 0.1
3	7.2 ± 0.1	60.1 ± 0.3	775 ± 4	5589 ± 184	1369 ± 42	55.3 ± 1.8	0.5 ± 0.5	3.0 ± 0.5
4	7.6 ± 0.1	55.2 ± 0.2	726 ± 6	5927 ± 276	1764 ± 66	126.3 ± 3.2	3.9 ± 0.4	2.3 ± 0.3
5	7.8 ± 0.0	55.6 ± 0.3	697 ± 7	7224 ± 160	1678 ± 212	108.7 ± 15.7	0.7 ± 0.0	4.7 ± 1.3
Dec-17								
1	5.6 ± 0.1	38.8 ± 0.8	827 ± 6	5872 ± 116	1292 ± 135	22.0 ± 2.1	26.9 ± 4.1	5.3 ± 0.4
2	7.0 ± 0.0	31.8 ± 1.6	701 ± 8	2912 ± 77	1931 ± 89	117.7 ± 4.7	77.9 ± 31.5	4.2 ± 0.9

3	7.4 ± 0.0	47.5 ± 1.7	774 ± 4	3945 ± 241	1198 ± 49	45.0 ± 0.6	36.9 ± 2.7	3.0 ± 0.1
4	7.6 ± 0.1	40.7 ± 0.6	741 ± 3	4040 ± 111	1524 ± 114	113.7 ± 4.3	47.7 ± 1.9	3.6 ± 0.4
5	7.7 ± 0.0	47.1 ± 0.4	705 ± 16	4784 ± 58	1728 ± 53	86.0 ± 1.7	1.6 ± 0.2	4.4 ± 0.5
Feb-18								
1	5.2 ± 0.1	34.7 ± 0.3	822 ± 4	9841 ± 1452	1063 ± 78	23.7 ± 3.5	30.6 ± 2.8	4.0 ± 0.3
2	6.7 ± 0.0	31.1 ± 0.7	674 ± 2	6623 ± 326	2080 ± 146	132.0 ± 7.5	7.8 ± 0.5	1.9 ± 0.1
3	7.0 ± 0.1	52.3 ± 3.3	737 ± 22	6484 ± 899	1013 ± 528	91.7 ± 32.3	0.5 ± 0.5	3.0 ± 0.5
4	7.3 ± 0.1	40.7 ± 4.5	708 ± 10	7963 ± 395	1089 ± 465	123.7 ± 3.8	3.9 ± 0.4	2.3 ± 0.3
5	7.7 ± 0.0	47.6 ± 1.2	696 ± 22	10983 ± 238	1782 ± 161	107.0 ± 7.6	0.7 ± 0.0	4.7 ± 1.3
Apr-18								
1	5.4 ± 0.2	35.1 ± 2.8	812 ± 4	7416 ± 568	1235 ± 124	21.7 ± 2.7		
2	6.6 ± 0.1	46.4 ± 0.4	685 ± 2	3828 ± 67	1988 ± 56	121.7 ± 7.4		
3	7.0 ± 0.0	50.9 ± 3.0	756 ± 4	5105 ± 404	1219 ± 10	54.0 ± 3.2		
4	7.3 ± 0.0	54.8 ± 0.2	711 ± 5	4981 ± 248	1582 ± 39	122.0 ± 2.6		
5	7.7 ± 0.0	52.5 ± 0.6	694 ± 10	6536 ± 158	1734 ± 52	124.3 ± 15.0		

Supplementary Table 2. Summary of RDA of major factors contributing to the changes in species compositions using two different primer pairs

Environmental variables	Explains (%)	Contribution (%)	pseudo-F	*P*
**515F-806R**				
pH	63.7	85.4	124	0.002
Organic matter	3.0	4.1	6.4	0.002
Dissolved organic carbon	2.7	3.6	6.1	0.002
Available phosphorus	1.2	1.6	2.8	0.004
Nitrate	1.2	1.6	2.9	0.01
Total phosphorus	1.1	1.5	2.8	0.008
Moisture	0.9	1.2	2.2	0.012
Ammonium	0.7	0.9	1.7	0.122
**515F-926R**				
pH	55.9	80.3	90.2	0.002
Dissolved organic carbon	3.7	5.4	7.0	0.002
Available phosphorus	3.6	5.2	6.3	0.002
Moisture	1.9	2.8	3.8	0.002
Nitrate	1.5	2.1	3.0	0.002
Total phosphorus	1.4	2.0	2.9	0.004
Ammonium	0.8	1.2	1.7	0.086
Organic matter	0.7	1.0	1.5	0.098

Supplementary Figures

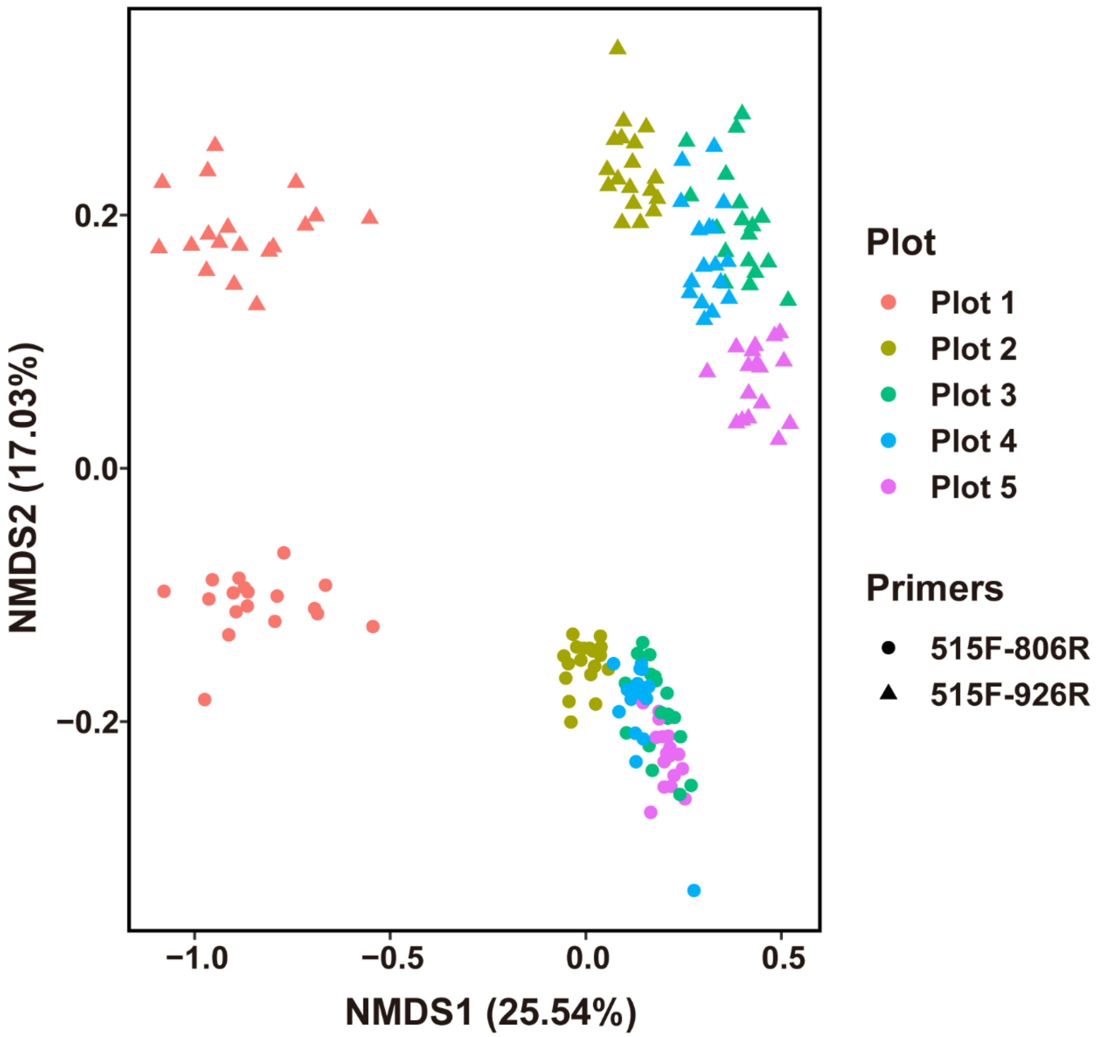

Supplementary Figure 1. Non-metric multi-dimensional scaling (NMDS) plot of the composition of total prokaryotic species in the five different plots by using primer pairs of 515F-806R or 515F-926R.

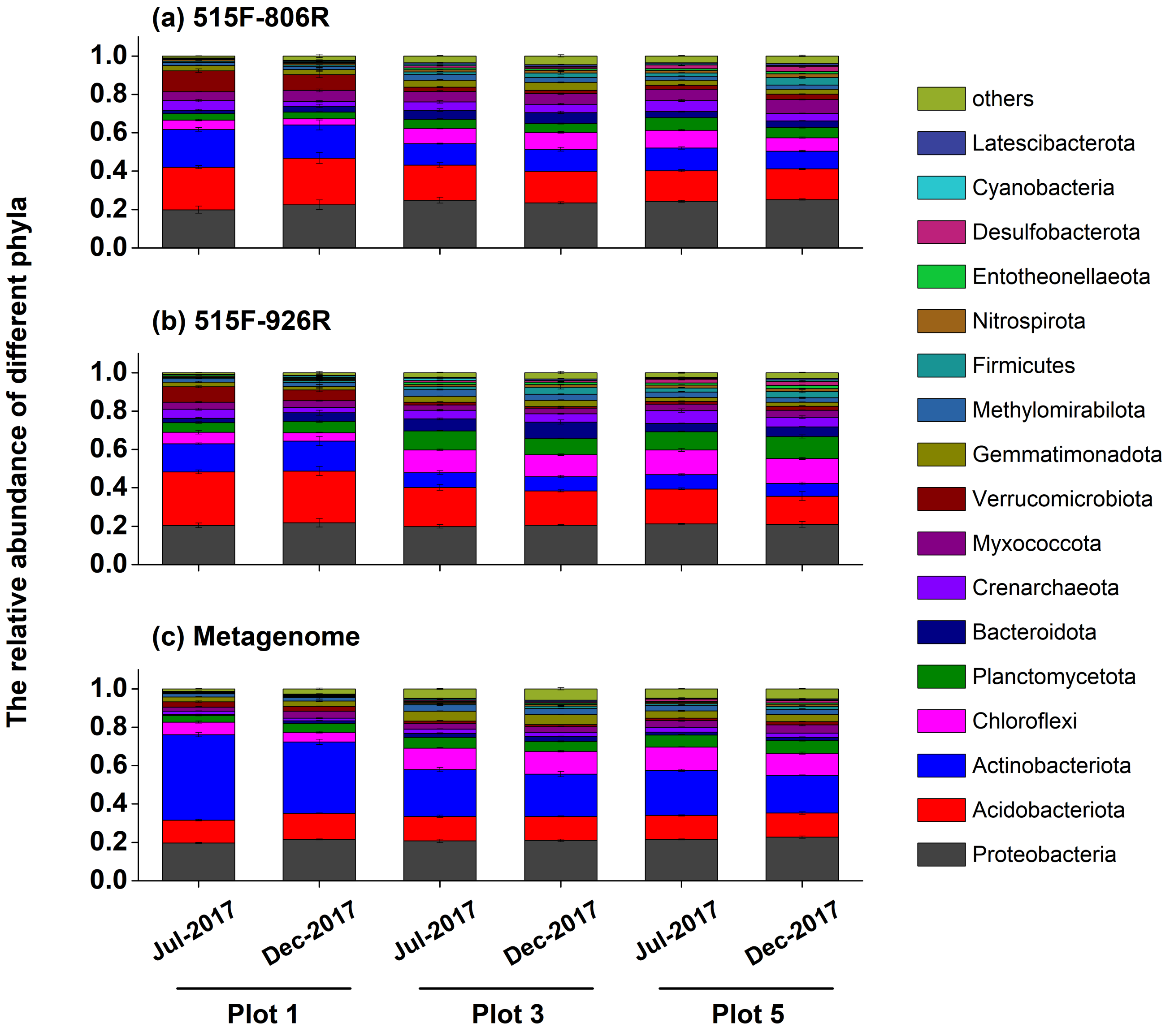

Supplementary Figure 2. The relative abundance of 17 major prokaryotic phyla (proportion > 0.5%) in soils. All the proportions of other phyla were summed and referred to as “others”. Error bars represent standard errors of means from triplicate samples.

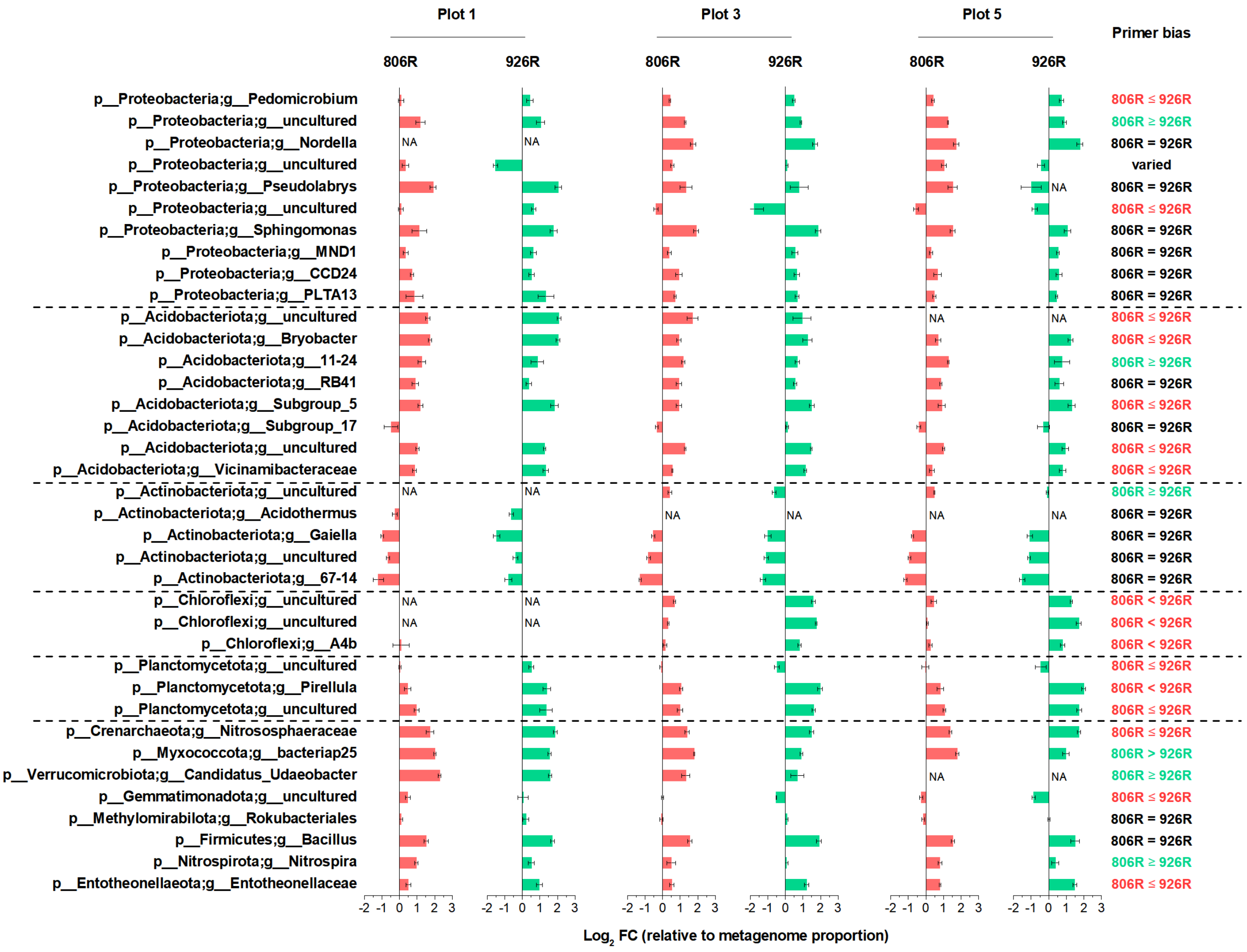

Supplementary Figure 3. The log2-transformed fold change in amplicon-based sequencing results (using 515F-806R or 515F-926R) relative to metagenome-derived 16S rRNA genes. Labels on the right column show generalization of primer bias as compared to metagenome-derived 16S rRNA genes. "806R < 926R" and "806R ≤ 926R" indicate 515F-806R results have less bias and more accurately estimate the proportion of the genera; "806R > 926R" or "806R ≥ 926R" indicate 515F-926R results have less bias and more accurately estimate the proportion of the genera; "806R = 926R" means both primer pairs have same degree of bias towards the genera; and "varied" means bias is not consistent in different soils.
